# Effectiveness Observation of Conjunctival Flap Transposition Combined With Placement of Lacrimal Ducts for the Repair of Eyelid Tumor Excision Involving the Lacrimal Point

**DOI:** 10.1155/joph/4709728

**Published:** 2025-06-24

**Authors:** Zhiyun Zhan, Jingjin Zhang, Enna Huang, Qiang Qu, Ting Wang, Tingting Wang

**Affiliations:** ^1^Department of Ophthalmology, The First Affiliated Hospital of Fujian Medical University, Fuzhou 350005, Fujian, China; ^2^Department of Ophthalmology, National Regional Medical Center, Binhai Campus of the First Affiliated Hospital, Fujian Medical University, Fuzhou 350005, Fujian, China; ^3^Fujian Institute of Ophthalmology, The First Affiliated Hospital of Fujian Medical University, Fuzhou 350005, Fujian, China; ^4^Fujian Provincial Clinical Medical Research Center of Eye Diseases and Optometry, The First Affiliated Hospital of Fujian Medical University, Fuzhou 350005, Fujian, China; ^5^Fujian Children's Hospital (Fujian Branch of Shanghai Children's Medical Center), Fuzhou 350005, Fujian, China

**Keywords:** conjunctival flap transposition, eyelid tumor, lacrimal point, melanocytic nevus

## Abstract

**Background:** Surgical excision of tumors near the lacrimal punctum presents challenges due to the risk of damaging the lacrimal duct, leading to chronic epiphora. Effective reconstruction is essential to preserve lacrimal function and maintain esthetic outcomes. This study discusses the short- to mid-term results of nine cases using conjunctival flap transposition and artificial lacrimal duct placement for repair, considering the duct's anatomical and functional aspects.

**Case Report:** We report on nine patients with benign lesions involving the lacrimal punctum at our hospital from August 2019 to September 2022. A conjunctival flap with a reasonable design based on the defect area was used to cover the defect, and double-tube placement of artificial lacrimal ducts was performed along with suturing of the remaining lacrimal point and lacrimal canaliculus epithelium. Pathological examination of all nine patients after surgery showed “melanocytic nevus.” The conjunctival flap survived in the first phase, the lacrimal point remained unblocked, there were no abnormalities in the lower eyelid or medial canthus, the appearance was aesthetically pleasing, lacrimal duct irrigation was unobstructed, and there was no tearing after surgery. All patients were followed up for more than 1 year, and no tumor recurrence or obstruction in the lacrimal duct was observed.

**Conclusions:** Conjunctival flap transposition and artificial lacrimal duct placement is an effective surgical approach for the repair of defects following lacrimal punctum tumor excision. The method not only preserves lacrimal duct function but also achieves satisfactory esthetic results, making it a reliable choice for clinical application.

**Summary**

• Repairing lacrimal punctal tumors which involve the artificial lacrimal tube placement together with transposition conjunctival flap after excision of perilacrimal tumors and lacrimal duct defects < 1/3 is reliable and a straightforward surgical option with high success rates.

## 1. Introduction

The eyelids are divided into upper and lower eyelids, which are soft tissues located in front of the eyeball. They are not only important accessory organs for protecting the eyeball but also significant esthetic symbols, playing a role in maintaining the moisture of the eyeball, blocking foreign objects, and maintaining tear drainage. The lacrimal punctum is located on the inner side of the upper and lower eyelids. Lesions around the lacrimal punctum are rare in ophthalmology and often present as round or annular small swellings around the lacrimal punctum. Currently reported lesions include nevus, epithelial inclusion cyst, nonpigmented compound nevus, papilloma, granuloma, and eosinophilic adenoma [1, 2].

Lesions around the lacrimal punctum not only affect aesthetics but also can invade and compress the lacrimal punctum, affecting tear absorption function and causing both psychological and physical discomfort to patients. The most effective method for removing lesions around the lacrimal punctum is surgical excision. After the surgical excision of lesions around the lacrimal punctum, along with the formation of the eyelid wound, there may be partial lacrimal duct loss, which adversely affects the lacrimal duct and leads to epiphora. In severe cases, the large lesions can damage the lacrimal canaliculi and cause lacrimal duct obstruction [3].

Therefore, after excising lesions around the lacrimal punctum, especially for larger lesions, effective reconstruction is essential to avoid complications such as epiphora and lacrimal duct obstruction. This study summarizes the clinical experience with nine patients treated from 2019 to 2022, involving conjunctival flap transposition and silicone stent placement for lacrimal duct reconstruction. All patients underwent tumor excision with concurrent lacrimal duct repair to ensure satisfactory postoperative outcomes.

## 2. Case Presentation

**Figure 1:**
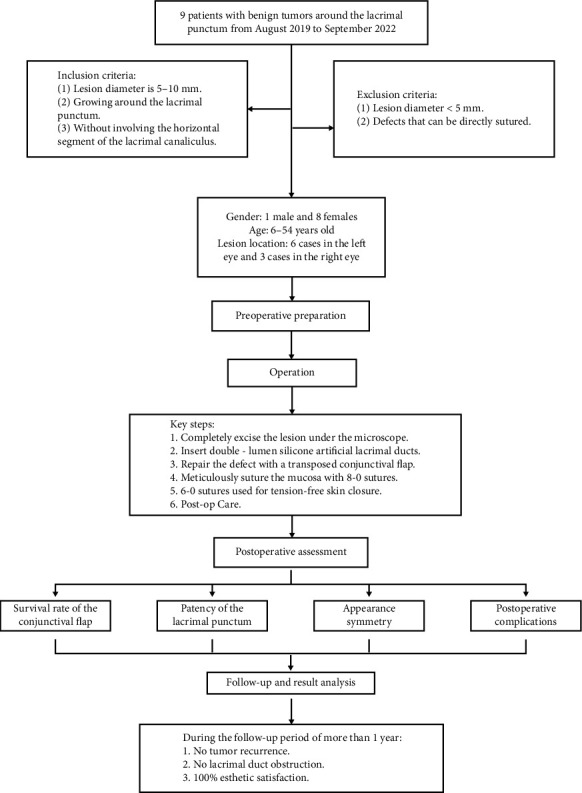
Flowchart of the report.

From September 2019 to September 2022, the Ophthalmology Department of the First Affiliated Hospital of Fujian Medical University treated nine cases of lesions around the lacrimal punctum. The cohort included one male and eight females, aged 6–54 years. Six cases were on the left side, and three cases were on the right side. The lesions were approximately circular or irregular in shape, with pigmentation on the surface, and with diameters ranging from 5 to 10 mm. Lesions smaller than 5 mm in diameter or those not encircling the lacrimal punctum, thus suitable for direct closure after excision, were not included in this study. This study was approved by the Ethics Committee of the First Affiliated Hospital of Fujian Medical University. The flowchart is shown in Figure 1.

## 3. Surgical Methods

Preoperative preparation: Preoperative assessment included routine evaluation to exclude surgical contraindications. Close-up external photographs and lacrimal punctum images of the patients were taken before the surgery.

Surgical methods: The procedure involved precise excision of the lesion and affected lacrimal punctum. Preoperatively, the extent of the lesion was thoroughly evaluated, ensuring complete removal of the lesion along its perimeter. Standard sterile preparation and draping were performed, followed by local infiltration anesthesia using 2% lidocaine to ensure adequate anesthesia of the operative field and surrounding tissues. Under an operating microscope, an incision was designed along the lesion margin. The skin and conjunctiva were incised vertically, and fine forceps were used to gently dissect the lesion layer by layer along its base to ensure complete removal. The excised tissue was sent for pathological examination.

In cases where the lesion involved partial loss of the lacrimal punctum or canaliculus, artificial lacrimal duct reconstruction was performed. A bicanalicular silicone tube (with metal guide probes) was inserted through both the upper and lower puncta, passing through the canaliculi, lacrimal sac, and nasolacrimal duct, with both ends emerging from the inferior nasal meatus. After confirming proper positioning and patency, the two ends were tied and fixed within the nasal cavity, avoiding the nasal vestibule to minimize discomfort and prevent dislodgement.

Based on the extent of the tissue defect, a conjunctival or sliding flap of appropriate size was designed to cover the area of lacrimal reconstruction. The flap was sutured to the surrounding mucosa of the residual punctum or canaliculus using 8-0 absorbable polyglactin sutures, ensuring mild eversion of the mucosal edge to facilitate tear drainage and epithelialization. For cases with significant skin defects lateral to the punctum, the wound margins were adequately mobilized and closed using 6-0 nonabsorbable polypropylene sutures, ensuring tension-free closure.

Postoperatively, a pressure dressing was applied. Skin sutures were typically removed on postoperative Day 7, and the silicone tube was removed within 4–12 weeks post-surgery.

## 4. Outcome

**Figure 2:**
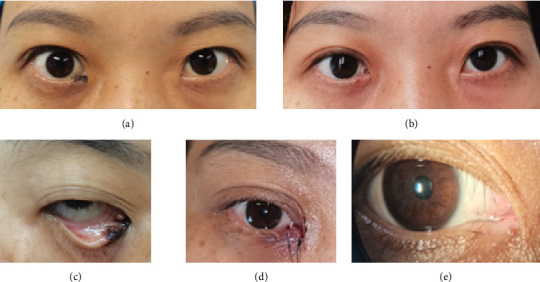
Case 1: (a) preoperative external photograph; (b) 3 months postoperation with artificial lacrimal duct; (c) photograph near the lacrimal punctum before the operation; (d) photograph near the lacrimal punctum 1 week postoperation; (e) photograph near the lacrimal punctum 8 months postoperation.

**Figure 3:**
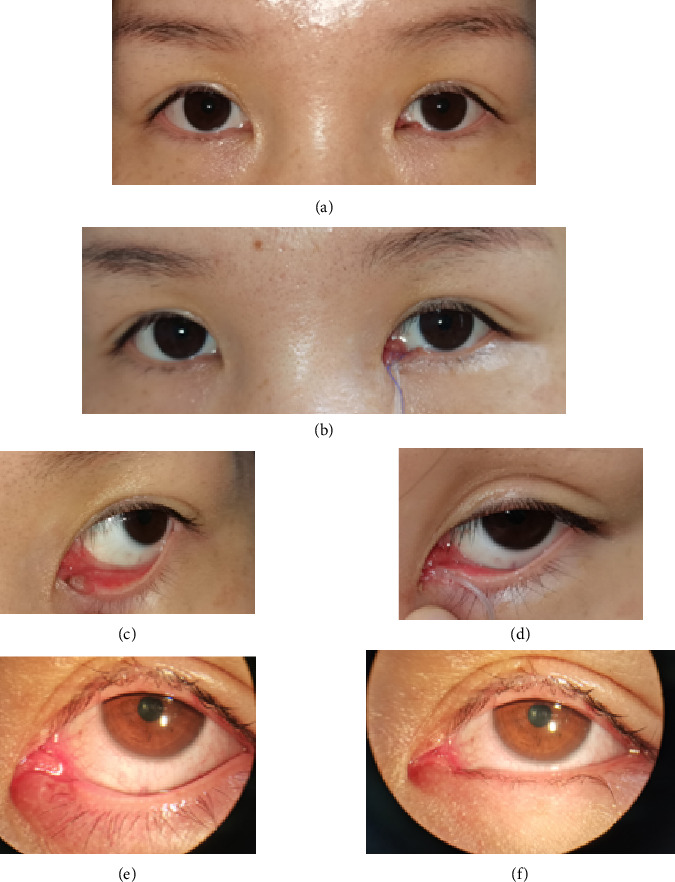
Case 2: (a) preoperative external photograph of the left eye; (b) 3 days postoperation with artificial lacrimal tube; (c) photograph near the lacrimal punctum before the operation; (d) photograph near the lacrimal punctum 1 month postoperation; (e) photograph near the lacrimal punctum 6 years postoperation (clean excision of the lesion and new lacrimal punctum opening good); (f) photograph near the lacrimal punctum 6 years postoperation in the natural state.

**Figure 4:**
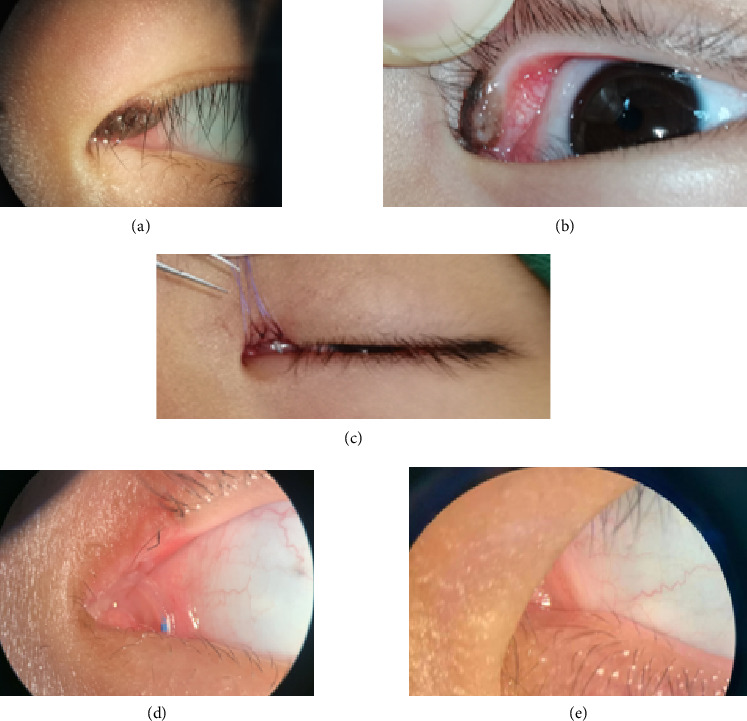
Case 3: (a) preoperative external photograph of the left eye; (b) preoperative photograph of the lesion and lacrimal punctum of the left eye; (c) immediate postoperative external photograph; (d) photograph near the lacrimal punctum 1 month postoperation with the tube in place; (e) natural state external photograph 3 months after tube removal.

The postoperative pathological examination of all nine patients revealed benign melanocytic nevi involving the lacrimal punctum. All patients underwent conjunctival flap combined with artificial lacrimal tube placement to repair the wound after excision of the lesion around the lacrimal punctum. The postoperative conjunctival flap and residual mucous membrane of the lacrimal punctum healed well. The morphology of the medial canthus was good compared to the healthy side after the operation, and the eyes were basically symmetrical. The lacrimal punctum was open, and the tear duct flushing was unobstructed. Over 1 year of follow-up, it showed no recurrence, canalicular obstruction, or adverse effects such as epiphora, ectropion, or entropion. Figures 2, 3, and 4 show the preoperative, postoperative, and postoperative follow-up photographs of the three patients, respectively.

## 5. Discussion

Lacrimal punctal tumors are classified into punctal adjacent tumors and peripunctal tumors, with the latter being less common, accounting for approximately 6%-7% of lacrimal punctal tumors. They can be categorized as benign or malignant. Benign tumors include nevus, keratinizing cysts, epithelial inclusion cysts, combined nevi, papillomas, pyogenic granulomas, and eosinophilic granulomas, while malignant tumors include basal cell carcinoma, malignant melanoma, and others [4]. In cases where the peripunctal tumors are large or malignant, the extent of removal often includes the punctum, the vertical segment of the canaliculus, and even parts of the horizontal segment and surrounding eyelid tissues. This may require further reconstruction of the eyelid alongside lacrimal duct formation. In the reported nine cases, all patients had benign tumors, and the excision range included parts or the entire vertical segment of the canaliculus, where the excised portion was less than one-third of the length of the canaliculus.

The perilacrimal growth of eyelid tumors presents in ophthalmology as a rare condition. It often manifests as circular or ring-shaped small swollen masses around the lacrimal punctum. As the growth of the tumor affects the lacrimal punctum, it invades and compresses the lacrimal drainage system, resulting not only in a cosmetic impact but also affecting the discharge function of the lacrimal pump, leading to epiphora causing patient psychological discomfort and life disturbances. Surgical principles for eyelid tumors involve complete excision of the tumor, with an adequate range and depth, followed by repairing the defect. When tumors involve the lacrimal punctum or canaliculi, simultaneous tumor excision, eyelid reconstruction, and lacrimal duct repair are recommended. Intraoperative damage to the lacrimal anatomy can result in punctal occlusion, canalicular stenosis, or postoperative obstruction, causing persistent epiphora [5]. While observation may be appropriate for small, asymptomatic benign lesions, surgery should be considered when tumors affect appearance, show progressive growth, or disrupt tear drainage. However, clinical studies on combined punctal excision and lacrimal reconstruction remain limited, and further investigation is warranted.

Our study demonstrates that for cases involving benign melanocytic nevi around the lacrimal punctum with canalicular defects of less than one-third, reconstruction using conjunctival flap transposition combined with artificial lacrimal duct stenting is a reliable and technically straightforward procedure. When performed with attention to key operative details, it yields favorable clinical outcomes. In contrast, reconstructive techniques for larger full-thickness lower eyelid defects, such as the MARCH technique, are primarily designed for extensive tissue loss. These procedures typically involve marginal incision, tissue mobilization, and tension-controlled closure, making the surgical process relatively complex and associated with a longer recovery period [6]. Such techniques offer clear advantages in structural reconstruction and are of significant clinical value in managing deep or full-thickness defects [7]. The surgical approach described in this study, however, is more suitable for localized and superficial defects around the lacrimal punctum. It preserves physiological tear drainage while also achieving favorable functional and esthetic outcomes, making it a viable option for selected indications.

This study has several limitations. Firstly, the sample size was relatively small, which may limit the generalizability of the findings. Secondly, all cases involve benign melanocytic nevi; thus, the applicability of this technique to malignant tumors or more extensive defects requires further investigation. Finally, although the follow-up period exceeded 1 year, long-term outcomes are still needed to evaluate the durability and stability of the reconstruction.

## 6. Conclusion

Conjunctival flap transposition combined with artificial lacrimal duct placement provides a reliable and straightforward surgical option for the repair of localized peripunctal defects following tumor excision. This approach preserves physiological tear drainage, achieves favorable functional and esthetic outcomes, and may serve as a practical treatment strategy for selected indications.

## Data Availability

The data used to support the findings of this study are available from the corresponding author upon request.
